# How to Integrate Echocardiographic Risk Factors for Atrial Fibrillation Following Acute Myocardial Infarction

**DOI:** 10.1002/clc.70163

**Published:** 2025-06-02

**Authors:** Naoya Kataoka, Teruhiko Imamura

**Affiliations:** ^1^ Second Department of Internal Medicine University of Toyama Toyama Japan

**Keywords:** ablation, electrocardiogram, left atrium

To Editor,

1

De novo atrial fibrillation (AF) following cardiac interventions frequently recurs, and early detection is particularly crucial in patients with acute myocardial infarction (AMI). The authors of the present study demonstrated that several echocardiographic parameters reflecting left atrial function were associated with the subsequent development of AF following AMI [1]. However, several concerns merit discussion.

Defining true de novo AF can be inherently challenging, as asymptomatic or subclinical AF may go undetected. How did the authors exclude the possibility of pre‐existing silent AF before the onset of AMI? Such undiagnosed episodes may have contributed to the observed left atrial remodeling.

The study population was limited to patients with AMI [1], in whom AF development is often precipitated by systemic inflammation or left atrial ischemia [2, 3]. Did the authors identify any supportive evidence for these mechanisms? For example, occlusion of the right coronary artery or left circumflex artery—both of which may involve atrial branches—could plausibly be linked to AF onset.

From a practical standpoint, how might these findings be applied in real‐world clinical settings? Even if we succeed in identifying patients at high risk, continuous rhythm monitoring using standard modalities would still be required for AF detection. Alternatively, could prophylactic catheter ablation be considered in select high‐risk individuals? Additionally, given that patients routinely receive antiplatelet therapy after AMI, the timing and feasibility of left atrial appendage closure following AF detection warrant further clarification [4].

## Ethics Statement

The authors have nothing to report.

## Consent

The authors have nothing to report.

## Conflicts of Interest

The authors declare no conflicts of interest.

## Data Availability

Data sharing not applicable to this article as no data sets were generated or analyzed during the current study.
